# Crystal structure of Al_8.77_Fe_0.80_Ni_1.20_Si_0.23_

**DOI:** 10.1107/S241431462600266X

**Published:** 2026-03-19

**Authors:** Mei Chen, Changzeng Fan, Bin Wen, Lifeng Zhang

**Affiliations:** a State Key Laboratory of Metastable Materials Science and Technology Yanshan University,Qinhuangdao 066004 People’s Republic of China; b Hebei Key Lab for Optimizing Metal Product Technology and Performance Yanshan University,Qinhuangdao 066004 People’s Republic of China; c School of Mechanical and Materials Engineering North China University of Technology,Beijing 100144 People’s Republic of China; Benemérita Universidad Autónoma de Puebla, México

**Keywords:** crystal structure, high-pressure, inter­metallic, quaternary system

## Abstract

The Al_8.77_Fe_0.80_Ni_1.20_Si_0.23_ (aluminium iron nickel silicate) phase, obtained *via* high-pressure sinter­ing of an Al-rich prealloy (nominal composition Al_78.08_Fe_8.65_Ni_8.69_Si_4.58_), is characterized as a novel phase in the Al—Si—Ni—Fe quaternary system. The obtained phase crystallizes in the space group *P*2_1_/*c*, with lattice parameters *a* = 6.2093 (9), *b* = 6.2579 (9), *c* = 8.5661 (12) Å, and *β* = 94.877 (5)°. It is isotypic with Al_9_Fe_0.7_Ni_1.28_ [*a* = 6.2406 (1), *b* = 6.2993 (1), *c* = 8.5992 (1) Å, and *β* = 95.129 (1)°; Chumak *et al.* (2007 ▸). *Inter­metallics*, **15**, 1416–1424] and Co_2_Al_9_ [*a* = 6.2163 (3), *b* = 6.2883 (3), *c* = 8.5587 (3) Å, and *β* = 94.772 (4)°; Boström *et al.* (2005 ▸). *Z. Anorg. Allg. Chem*. **631**, 534–541]. It features a co-occupancy of Al and Si atoms with a ratio of the refined site-occupancy factors of 0.88 (10):0.12 (10), as well a co-occupancy Ni/Fe with site-occupancy factors of 0.60 (4):0.40 (4).

## Abstract 2

The Al_8.77_Fe_0.80_Ni_1.20_Si_0.23_ (aluminium iron nickel silicate) phase, obtained *via* high-pressure sinter­ing of an Al-rich prealloy (nominal composition Al_78.08_Fe_8.65_Ni_8.69_Si_4.58_), is characterized as a novel phase in the Al—Si—Ni—Fe quaternary system.

## Graphical abstract

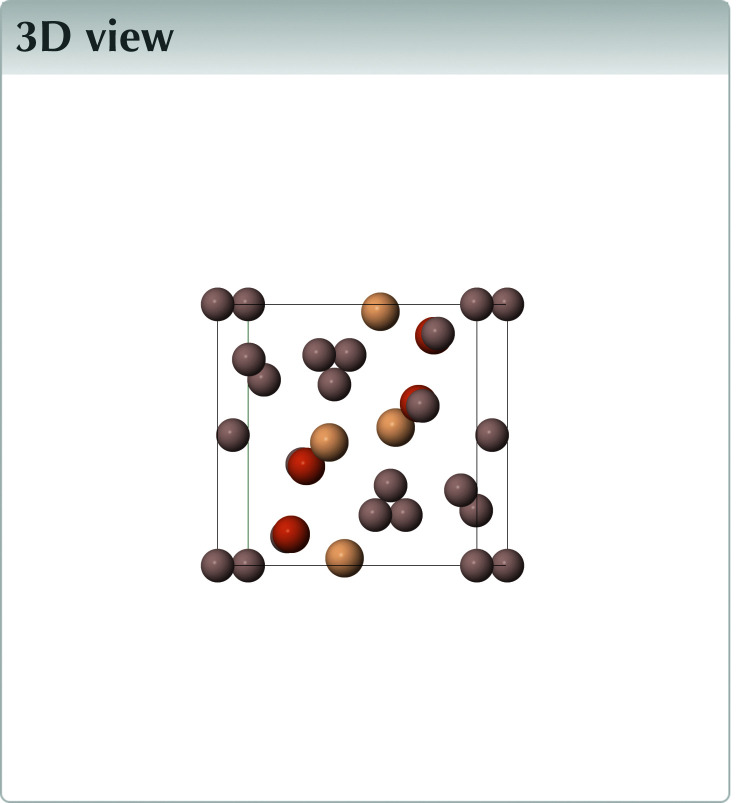

## Structure description

**Figure 1:**
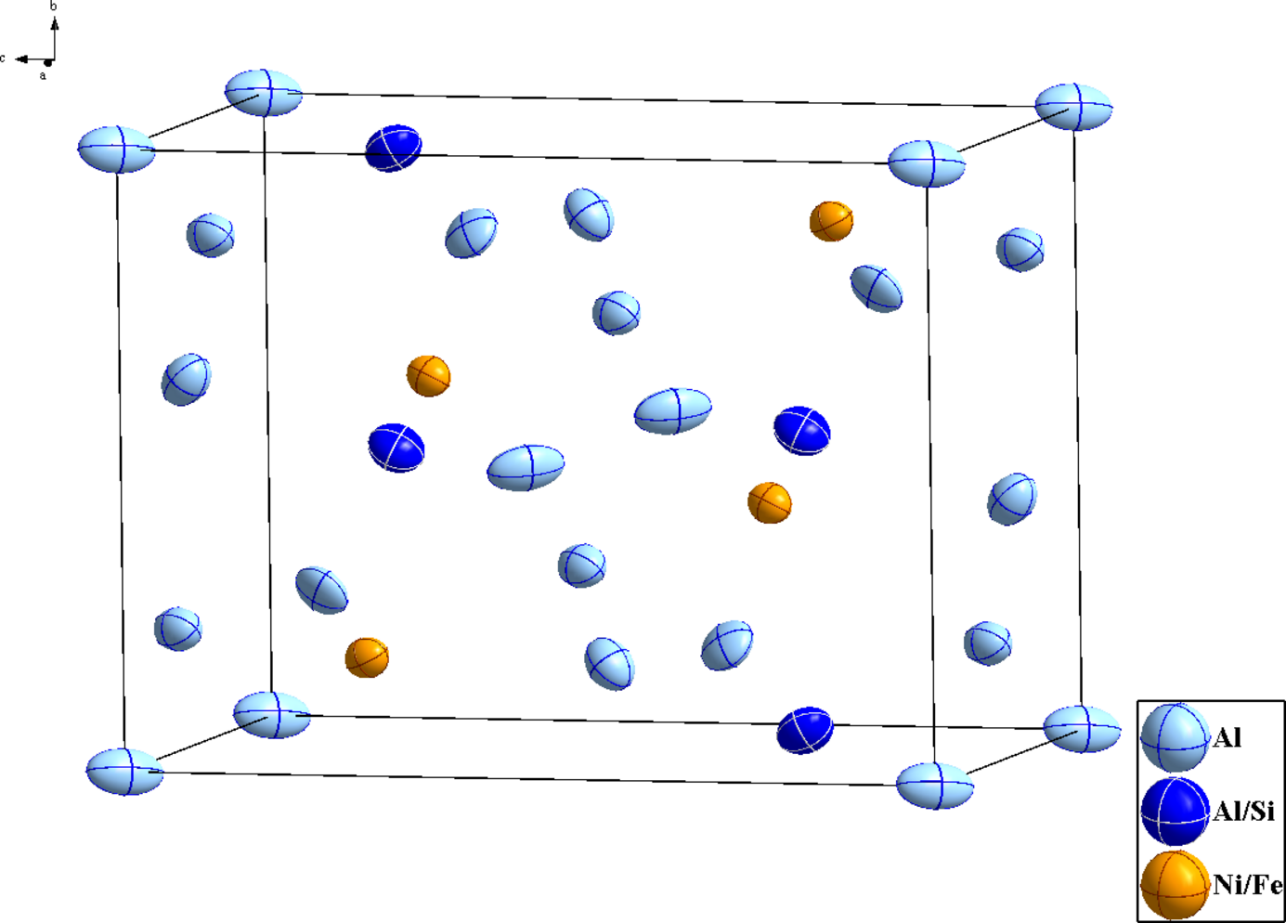
The crystal structure of Al_8.77_Fe_0.80_Ni_1.20_Si_0.23_ (one unit cell), with displacement ellipsoids drawn at the 90% probability level.

**Figure 2:**
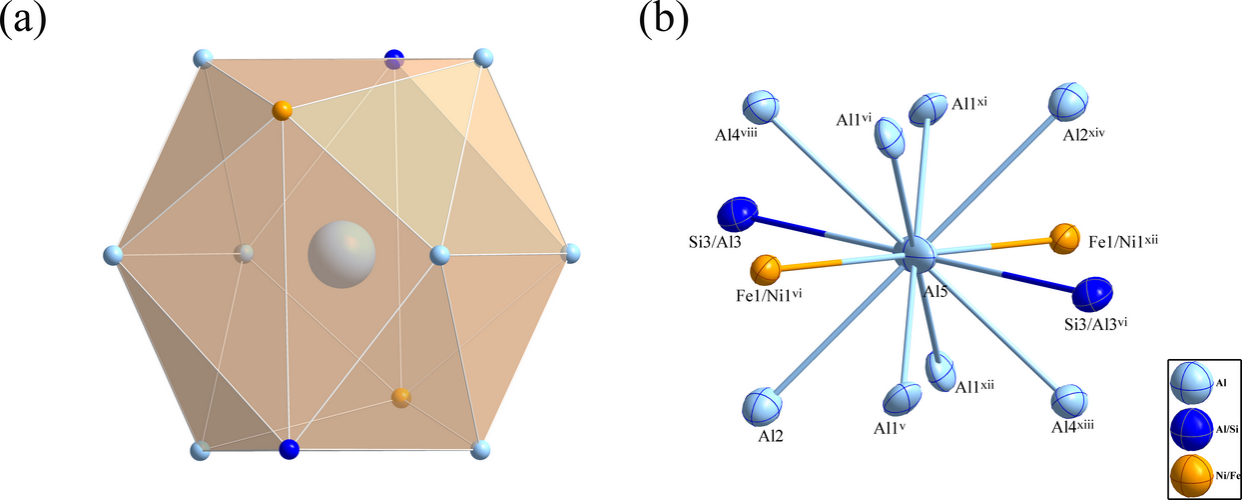
(*a*) The icosa­hedron formed around the Al5 atom at the 2*a* site and (*b*) the environment of the Al5 atom, with displacement ellipsoids given at the 90% probability level. [Symmetry codes: (v) −*x*, −*y* + 1, −*z*; (vi) *x*, −*y* + 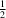, *z* − 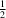; (viii) −*x* + 1, −*y*, −*z*; (xi) *x*, *y* − 1, *z*; (xii) −*x*, *y* − 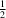, −*z* + 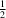; (xiii) *x* − 1, *y*, *z*; (xiv) −*x*, −*y*, −*z*.]

It has been reported that the solubility of Si in the Al_9_FeNi phase is approximately 4 wt% (Belov *et al.*, 2002 ▸), and this conclusion has been further validated by subsequent experimental investigations (Hao *et al.*, 2014 ▸). In the current work, the nominal composition of the inter­metallic compound Al_78.08_Fe_8.65_Ni_8.69_Si_4.58_ was designed on the basis of the reported Si solubility (4 wt%) in the Al_9_FeNi phase. Via high-pressure sinter­ing, laboratory experiments were carried out to investigate the formation behaviour of this phase; consequently, a crystalline inter­metallic phase with a composition of Al_8.77_Fe_0.80_Ni_1.20_Si_0.23_ was successfully obtained. This phase shows remarkable structural similarities to Al_9_Fe_0.7_Ni_1.3_ [*a* = 6.2406 (1), *b* = 6.2993 (1), *c* = 8.5992 (1) Å, and *β* = 95.129 (1)°] reported by Chumak *et al.* (2007 ▸), sharing identical space-group symmetry and analogous co-site occupancy characteristics. Al_8.77_Fe_0.80_Ni_1.20_Si_0.23_, along with Al_9_Fe_0.7_Ni_1.3_ and other *T*_2_Al_9_-type compounds (*T* = Co, Rh, Ir), crystallizes in the space group *P*2_1_/*c* (No. 14). The atomic distribution within the unit cell of Al_8.77_Fe_0.80_Ni_1.20_Si_0.23_ is illustrated in Fig. 1 ▸. The environment of atom Al5 is shown in Fig. 2 ▸. It is located at special position 2*a* (inversion centre) and is coordinated by 12 atoms, forming the centre of a distorted icosa­hedron.

In this study, we refined the crystal structure model of Al_8.77_Fe_0.80_Ni_1.20_Si_0.23_ based on single-crystal X-ray diffraction data. Its composition was confirmed by EDX results (see the supporting information).

## Synthesis and crystallization

High-purity aluminium (indicated purity 99.9%; 0.6528 g), iron (indicated purity 99.9%; 0.1516 g), nickel (indicated purity 99.9%; 0.1579 g), and silicon (indicated purity 99.9%; 0.0416 g) with a stoichiometric ratio of 78.08:8.65:8.69:4.58 were evenly mixed and fully ground in an agate mortar for 40 min. The homogenized powder was placed in a boron nitride furnace die with a diameter of 5 mm, compacted with a small rod, and subsequently subjected to high-pressure sinter­ing using a six-anvil high-temperature and high-pressure apparatus. Cylindrical blocks without deformation and cracks were obtained. Details of high-pressure sinter­ing experiments using six-anvil high-temperature and high-pressure equipment are described elsewhere (Liu & Fan, 2018 ▸). The sample was pressurized to 6 GPa and heated to 1676 K for 30 min., then cooled to 1131 K and held for 60 min., and finally rapidly cooled to room temperature by turning off the furnace power. A single crystal (0.08 × 0.07 × 0.06 mm^3^) was selected and mounted on a glass fibre for measurements.

## Refinement

Crystal data, data collection and structure refinement details are summarized in Table 1 ▸. To facilitate comparative analysis, the labelling scheme and atomic coordinates for Al_8.77_Fe_0.80_Ni_1.20_Si_0.23_ were taken from the corresponding data for Al_9_Fe_0.7_Ni_1.3_ (Chumak *et al.*, 2007 ▸). The occupancy factors for Al3 and Si3 atoms sharing the same site were refined to 0.88 (10) and 0.12 (10); the occupancy factors for Ni1 and Fe1 atoms sharing the same site were refined to 0.60 (4) and 0.40 (4), respectively. The maximum and minimum residual electron densities in the final difference map are located 1.35 Å from Al4 and 0.92 Å from Al3/Si3, respectively.

## Supplementary Material

Crystal structure: contains datablock(s) I. DOI: 10.1107/S241431462600266X/bh4102sup1.cif

Structure factors: contains datablock(s) I. DOI: 10.1107/S241431462600266X/bh4102Isup2.hkl

EDX spectra (supplementary material). DOI: 10.1107/S241431462600266X/bh4102sup3.docx

CCDC reference: 2537011

Additional supporting information:  crystallographic information; 3D view; checkCIF report

## Figures and Tables

**Table 1:** Experimental details

Crystal data	
Chemical formula	Al_8.77_Fe_0.80_Ni_1.20_Si_0.23_
*M*_r_	358.20
Crystal system, space group	Monoclinic, *P*2_1_/*c*
Temperature (K)	296
*a*, *b*, *c* (Å)	6.2093 (9), 6.2579 (9), 8.5661 (12)
β (°)	94.877 (5)
*V* (Å^3^)	331.65 (8)
*Z*	2
Radiation type	Mo *K*α
μ (mm^−1^)	6.24
Crystal size (mm)	0.08 × 0.07 × 0.06

Data collection	
Diffractometer	Bruker D8 Venture Photon 100 CMOS
Absorption correction	Multi-scan (*SADABS*; Krause *et al.*, 2015 ▸)
*T*_min_, *T*_max_	0.599, 0.746
No. of measured, independent and observed [*I* > 2σ(*I*)] reflections	7812, 769, 606
*R*_int_	0.098
(sin θ/λ)_max_ (Å^−1^)	0.650

Refinement	
*R*[*F*^2^ > 2σ(*F*^2^)], *wR*(*F*^2^), *S*	0.037, 0.070, 1.08
No. of reflections	769
No. of parameters	54
Δρ_max_, Δρ_min_ (e Å^−3^)	0.73, −0.68

Computer programs: *APEX5* and *SAINT* (Bruker, 2023 ▸), *SHELXT* (Sheldrick, 2015*a* ▸), *SHELXL* (Sheldrick, 2015*b* ▸), *DIAMOND* (Brandenburg & Putz, 2017 ▸) and *publCIF* (Westrip, 2010 ▸).

## full crystallographic data

### Aluminium iron nickel silicate Crystal data

**Table:**

Al_8.77_Fe_0.80_Ni_1.20_Si_0.23_	*F*(000) = 343
*M**_r_* = 358.20	*D*_x_ = 3.587 Mg m^−^^3^
Monoclinic, *P*2_1_/*c*	Mo *K*α radiation, λ = 0.71073 Å
*a* = 6.2093 (9) Å	Cell parameters from 2687 reflections
*b* = 6.2579 (9) Å	θ = 3.3–26.8°
*c* = 8.5661 (12) Å	µ = 6.24 mm^−^^1^
β = 94.877 (5)°	*T* = 296 K
*V* = 331.65 (8) Å^3^	Lump, grey
*Z* = 2	0.08 × 0.07 × 0.06 mm

### Aluminium iron nickel silicate Data collection

**Table:**

Bruker D8 Venture Photon 100 CMOS diffractometer	606 reflections with *I* > 2σ(*I*)
phi and ω scans	*R*_int_ = 0.098
Absorption correction: multi-scan (SADABS; Krause *et al.*, 2015)	θ_max_ = 27.5°, θ_min_ = 3.3°
*T*_min_ = 0.599, *T*_max_ = 0.746	*h* = −8→8
7812 measured reflections	*k* = −8→8
769 independent reflections	*l* = −11→11

### Aluminium iron nickel silicate Refinement

**Table:**

Refinement on *F*^2^	0 restraints
Least-squares matrix: full	Primary atom site location: dual
*R*[*F*^2^ > 2σ(*F*^2^)] = 0.037	Secondary atom site location: difference Fourier map
*wR*(*F*^2^) = 0.070	*w* = 1/[σ^2^(*F*_o_^2^) + (0.0247*P*)^2^ + 0.9238*P*] where *P* = (*F*_o_^2^ + 2*F*_c_^2^)/3
*S* = 1.08	(Δ/σ)_max_ < 0.001
769 reflections	Δρ_max_ = 0.73 e Å^−^^3^
54 parameters	Δρ_min_ = −0.68 e Å^−^^3^

### Aluminium iron nickel silicate Fractional atomic coordinates and isotropic or equivalent isotropic displacement parameters (Å^2^)

**Table:**

	*x*	*y*	*z*	*U*_iso_*/*U*_eq_	Occ. (<1)
Al1	0.0887 (2)	0.7113 (2)	0.22978 (17)	0.0100 (4)	
Al2	0.2136 (2)	0.3882 (2)	0.04319 (17)	0.0103 (4)	
Al3	0.4038 (2)	0.0285 (2)	0.26814 (17)	0.0107 (6)	0.88 (10)
Si3	0.4038 (2)	0.0285 (2)	0.26814 (17)	0.0107 (6)	0.12 (10)
Al4	0.6089 (2)	0.1934 (2)	0.00355 (17)	0.0091 (4)	
Ni1	0.26441 (10)	0.37995 (10)	0.33345 (7)	0.0071 (2)	0.60 (4)
Fe1	0.26441 (10)	0.37995 (10)	0.33345 (7)	0.0071 (2)	0.40 (4)
Al5	0.000000	0.000000	0.000000	0.0140 (5)	

### Aluminium iron nickel silicate Atomic displacement parameters (Å^2^)

**Table:**

	*U*^11^	*U*^22^	*U*^33^	*U*^12^	*U*^13^	*U*^23^
Al1	0.0075 (8)	0.0099 (8)	0.0121 (8)	0.0032 (6)	−0.0011 (6)	0.0023 (6)
Al2	0.0095 (8)	0.0109 (8)	0.0104 (8)	0.0009 (6)	0.0006 (6)	−0.0026 (6)
Al3	0.0101 (9)	0.0095 (9)	0.0127 (10)	0.0018 (6)	0.0022 (6)	−0.0021 (6)
Si3	0.0101 (9)	0.0095 (9)	0.0127 (10)	0.0018 (6)	0.0022 (6)	−0.0021 (6)
Al4	0.0086 (7)	0.0077 (7)	0.0106 (8)	0.0005 (6)	−0.0010 (6)	−0.0001 (6)
Ni1	0.0060 (4)	0.0068 (4)	0.0085 (4)	−0.0005 (3)	−0.0002 (2)	0.0007 (3)
Fe1	0.0060 (4)	0.0068 (4)	0.0085 (4)	−0.0005 (3)	−0.0002 (2)	0.0007 (3)
Al5	0.0151 (11)	0.0088 (11)	0.0196 (12)	0.0002 (9)	0.0115 (9)	0.0017 (9)

### Aluminium iron nickel silicate Geometric parameters (Å, º)

**Table:**

Al1—Ni1^i^	2.4521 (16)	Al3—Ni1	2.4456 (15)
Al1—Fe1	2.4727 (16)	Al3—Ni1^vii^	2.4842 (15)
Al1—Ni1	2.4727 (16)	Al3—Al4^viii^	2.706 (2)
Al1—Al5^ii^	2.6938 (15)	Al3—Al4^vii^	2.873 (2)
Al1—Al2	2.730 (2)	Al3—Al4^ix^	2.878 (2)
Al1—Al5^i^	2.7618 (15)	Al3—Al4	2.884 (2)
Al1—Al3^ii^	2.787 (2)	Si3—Fe1	2.4456 (15)
Al1—Al4^iii^	2.833 (2)	Si3—Al4^viii^	2.706 (2)
Al1—Al4^iv^	2.918 (2)	Si3—Al4^vii^	2.873 (2)
Al1—Al2^v^	2.938 (2)	Si3—Al4^ix^	2.878 (2)
Al2—Fe1	2.4801 (17)	Si3—Al4	2.884 (2)
Al2—Ni1	2.4801 (17)	Al4—Ni1^vii^	2.4960 (16)
Al2—Ni1^vi^	2.4980 (16)	Al4—Ni1^vi^	2.5260 (16)
Al2—Al3^vi^	2.773 (2)	Al4—Al5^x^	2.7158 (15)
Al2—Al5	2.7774 (15)	Al4—Al4^viii^	2.772 (3)
Al2—Al4	2.787 (2)	Ni1—Al5^i^	2.3861 (7)
Al2—Al4^iv^	2.882 (2)	Fe1—Al5^i^	2.3861 (7)
Al2—Al3^iii^	2.894 (2)		
			
Ni1^i^—Al1—Ni1	143.05 (6)	Ni1^vii^—Al4—Al1^vii^	54.85 (4)
Ni1^i^—Al1—Al5^ii^	55.01 (3)	Ni1^vi^—Al4—Al1^vii^	160.56 (7)
Ni1—Al1—Al5^ii^	151.81 (6)	Al3^viii^—Al4—Al1^vii^	133.06 (7)
Ni1^i^—Al1—Al2	118.85 (7)	Al5^x^—Al4—Al1^vii^	59.65 (4)
Fe1—Al1—Al2	56.67 (5)	Al4^viii^—Al4—Al1^vii^	109.93 (8)
Ni1—Al1—Al2	56.67 (5)	Al2—Al4—Al1^vii^	114.50 (7)
Al5^ii^—Al1—Al2	96.86 (6)	Ni1^vii^—Al4—Al3^iii^	102.61 (6)
Ni1^i^—Al1—Al5^i^	98.79 (5)	Ni1^vi^—Al4—Al3^iii^	117.03 (6)
Fe1—Al1—Al5^i^	53.90 (3)	Al3^viii^—Al4—Al3^iii^	163.69 (7)
Ni1—Al1—Al5^i^	53.90 (3)	Al5^x^—Al4—Al3^iii^	114.16 (6)
Al5^ii^—Al1—Al5^i^	152.94 (6)	Al4^viii^—Al4—Al3^iii^	127.65 (8)
Al2—Al1—Al5^i^	102.88 (6)	Al2—Al4—Al3^iii^	61.48 (5)
Ni1^i^—Al1—Al3^ii^	108.97 (6)	Al1^vii^—Al4—Al3^iii^	58.46 (5)
Ni1—Al1—Al3^ii^	105.73 (6)	Ni1^vii^—Al4—Al3^vi^	165.23 (7)
Al5^ii^—Al1—Al3^ii^	72.93 (5)	Ni1^vi^—Al4—Al3^vi^	53.34 (4)
Al2—Al1—Al3^ii^	111.41 (6)	Al3^viii^—Al4—Al3^vi^	74.27 (4)
Al5^i^—Al1—Al3^ii^	115.49 (6)	Al5^x^—Al4—Al3^vi^	127.21 (6)
Ni1^i^—Al1—Al4^iii^	135.55 (7)	Al4^viii^—Al4—Al3^vi^	109.07 (8)
Ni1—Al1—Al4^iii^	55.63 (4)	Al2—Al4—Al3^vi^	58.60 (5)
Al5^ii^—Al1—Al4^iii^	134.11 (6)	Al1^vii^—Al4—Al3^vi^	139.88 (7)
Al2—Al1—Al4^iii^	103.95 (6)	Al3^iii^—Al4—Al3^vi^	89.98 (6)
Al5^i^—Al1—Al4^iii^	58.06 (4)	Ni1^vii^—Al4—Al2^iv^	133.30 (7)
Al3^ii^—Al1—Al4^iii^	61.49 (5)	Ni1^vi^—Al4—Al2^iv^	113.59 (6)
Ni1^i^—Al1—Al4^iv^	112.10 (6)	Al3^viii^—Al4—Al2^iv^	109.29 (6)
Ni1—Al1—Al4^iv^	97.32 (6)	Al5^x^—Al4—Al2^iv^	92.90 (5)
Al5^ii^—Al1—Al4^iv^	57.72 (4)	Al4^viii^—Al4—Al2^iv^	169.21 (9)
Al2—Al1—Al4^iv^	61.25 (5)	Al2—Al4—Al2^iv^	88.33 (6)
Al5^i^—Al1—Al4^iv^	149.07 (6)	Al1^vii^—Al4—Al2^iv^	80.86 (6)
Al3^ii^—Al1—Al4^iv^	56.57 (5)	Al3^iii^—Al4—Al2^iv^	57.62 (5)
Al4^iii^—Al1—Al4^iv^	98.02 (6)	Al3^vi^—Al4—Al2^iv^	60.33 (5)
Ni1^i^—Al1—Al2^v^	54.31 (4)	Ni1^vii^—Al4—Al3	54.43 (4)
Ni1—Al1—Al2^v^	109.91 (6)	Ni1^vi^—Al4—Al3	88.81 (5)
Al5^ii^—Al1—Al2^v^	58.91 (4)	Al3^viii^—Al4—Al3	120.65 (6)
Al2—Al1—Al2^v^	64.61 (6)	Al5^x^—Al4—Al3	107.72 (6)
Al5^i^—Al1—Al2^v^	113.96 (6)	Al4^viii^—Al4—Al3	57.12 (6)
Al3^ii^—Al1—Al2^v^	129.75 (7)	Al2—Al4—Al3	66.94 (5)
Al4^iii^—Al1—Al2^v^	165.48 (7)	Al1^vii^—Al4—Al3	71.77 (6)
Al4^iv^—Al1—Al2^v^	84.43 (6)	Al3^iii^—Al4—Al3	71.74 (4)
Ni1—Al2—Ni1^vi^	133.95 (6)	Al3^vi^—Al4—Al3	124.64 (7)
Fe1—Al2—Al1	56.41 (5)	Al2^iv^—Al4—Al3	129.35 (7)
Ni1—Al2—Al1	56.41 (5)	Al5^x^—Al4—Si3	107.72 (6)
Ni1^vi^—Al2—Al1	167.97 (7)	Al4^viii^—Al4—Si3	57.12 (6)
Ni1—Al2—Al3^vi^	145.96 (7)	Al2—Al4—Si3	66.94 (5)
Ni1^vi^—Al2—Al3^vi^	54.99 (4)	Al1^vii^—Al4—Si3	71.77 (6)
Al1—Al2—Al3^vi^	121.39 (7)	Al2^iv^—Al4—Si3	129.35 (7)
Fe1—Al2—Al5	97.70 (5)	Al5^i^—Ni1—Al3	134.01 (4)
Ni1—Al2—Al5	97.70 (5)	Al5^i^—Ni1—Al1^xii^	67.65 (4)
Ni1^vi^—Al2—Al5	53.47 (3)	Al3—Ni1—Al1^xii^	83.66 (5)
Al1—Al2—Al5	124.72 (6)	Al5^i^—Ni1—Al1	69.25 (4)
Al3^vi^—Al2—Al5	106.51 (6)	Al3—Ni1—Al1	145.68 (6)
Fe1—Al2—Al4	94.29 (6)	Al1^xii^—Ni1—Al1	85.56 (3)
Ni1—Al2—Al4	94.29 (6)	Al5^i^—Ni1—Al2	123.88 (4)
Ni1^vi^—Al2—Al4	56.78 (4)	Al3—Ni1—Al2	78.86 (5)
Al1—Al2—Al4	133.79 (7)	Al1^xii^—Ni1—Al2	75.66 (5)
Al3^vi^—Al2—Al4	62.33 (5)	Al1—Ni1—Al2	66.91 (5)
Al5—Al2—Al4	90.96 (5)	Al5^i^—Ni1—Al3^iii^	136.46 (4)
Ni1—Al2—Al4^iv^	98.08 (6)	Al3—Ni1—Al3^iii^	86.35 (3)
Ni1^vi^—Al2—Al4^iv^	115.93 (6)	Al1^xii^—Ni1—Al3^iii^	146.76 (6)
Al1—Al2—Al4^iv^	62.58 (5)	Al1—Ni1—Al3^iii^	85.07 (5)
Al3^vi^—Al2—Al4^iv^	61.04 (5)	Al2—Ni1—Al3^iii^	71.32 (5)
Al5—Al2—Al4^iv^	163.76 (6)	Al5^i^—Ni1—Al4^iii^	67.55 (4)
Al4—Al2—Al4^iv^	91.67 (6)	Al3—Ni1—Al4^iii^	137.22 (6)
Ni1—Al2—Al3^iii^	54.41 (4)	Al1^xii^—Ni1—Al4^iii^	134.03 (5)
Ni1^vi^—Al2—Al3^iii^	117.23 (6)	Al1—Ni1—Al4^iii^	69.52 (5)
Al1—Al2—Al3^iii^	73.08 (6)	Al2—Ni1—Al4^iii^	123.48 (5)
Al3^vi^—Al2—Al3^iii^	91.65 (6)	Al3^iii^—Ni1—Al4^iii^	70.77 (5)
Al5—Al2—Al3^iii^	134.41 (6)	Al5^i^—Ni1—Al2^ix^	69.27 (4)
Al4—Al2—Al3^iii^	60.72 (5)	Al3—Ni1—Al2^ix^	68.24 (5)
Al4^iv^—Al2—Al3^iii^	59.76 (5)	Al1^xii^—Ni1—Al2^ix^	72.81 (5)
Ni1—Al2—Al1^v^	144.26 (6)	Al1—Ni1—Al2^ix^	137.97 (5)
Ni1^vi^—Al2—Al1^v^	52.87 (4)	Al2—Ni1—Al2^ix^	136.22 (4)
Al1—Al2—Al1^v^	115.39 (6)	Al3^iii^—Ni1—Al2^ix^	131.50 (5)
Al3^vi^—Al2—Al1^v^	69.70 (5)	Al4^iii^—Ni1—Al2^ix^	100.29 (6)
Al5—Al2—Al1^v^	56.15 (4)	Al5^i^—Ni1—Al4^ix^	107.72 (4)
Al4—Al2—Al1^v^	108.67 (6)	Al3—Ni1—Al4^ix^	70.71 (5)
Al4^iv^—Al2—Al1^v^	107.94 (6)	Al1^xii^—Ni1—Al4^ix^	138.31 (6)
Al3^iii^—Al2—Al1^v^	161.34 (7)	Al1—Ni1—Al4^ix^	133.30 (5)
Ni1—Al3—Ni1^vii^	137.46 (7)	Al2—Ni1—Al4^ix^	127.69 (5)
Ni1—Al3—Al4^viii^	132.33 (7)	Al3^iii^—Ni1—Al4^ix^	65.36 (5)
Ni1^vii^—Al3—Al4^viii^	58.06 (4)	Al4^iii^—Ni1—Al4^ix^	66.99 (6)
Ni1—Al3—Al2^ix^	56.78 (4)	Al2^ix^—Ni1—Al4^ix^	67.39 (5)
Ni1^vii^—Al3—Al2^ix^	142.53 (7)	Al5^i^—Fe1—Si3	134.01 (4)
Al4^viii^—Al3—Al2^ix^	147.55 (7)	Al5^i^—Fe1—Al1^xii^	67.65 (4)
Ni1—Al3—Al1^xi^	114.14 (6)	Al5^i^—Fe1—Al1	69.25 (4)
Ni1^vii^—Al3—Al1^xi^	106.68 (6)	Si3—Fe1—Al1	145.68 (6)
Al4^viii^—Al3—Al1^xi^	64.16 (5)	Al1^xii^—Fe1—Al1	85.56 (3)
Al2^ix^—Al3—Al1^xi^	83.61 (6)	Al5^i^—Fe1—Al2	123.88 (4)
Ni1—Al3—Al4^vii^	118.03 (6)	Si3—Fe1—Al2	78.86 (5)
Ni1^vii^—Al3—Al4^vii^	92.11 (6)	Al1^xii^—Fe1—Al2	75.66 (5)
Al4^viii^—Al3—Al4^vii^	102.15 (5)	Al1—Fe1—Al2	66.91 (5)
Al2^ix^—Al3—Al4^vii^	61.35 (5)	Al5^i^—Fe1—Al4^iii^	67.55 (4)
Al1^xi^—Al3—Al4^vii^	60.05 (5)	Al1^xii^—Fe1—Al4^iii^	134.03 (5)
Ni1—Al3—Al4^ix^	55.95 (4)	Al1—Fe1—Al4^iii^	69.52 (5)
Ni1^vii^—Al3—Al4^ix^	98.09 (6)	Al2—Fe1—Al4^iii^	123.48 (5)
Al4^viii^—Al3—Al4^ix^	153.13 (6)	Al5^i^—Fe1—Al2^ix^	69.27 (4)
Al2^ix^—Al3—Al4^ix^	59.07 (5)	Al1^xii^—Fe1—Al2^ix^	72.81 (5)
Al1^xi^—Al3—Al4^ix^	141.07 (7)	Al1—Fe1—Al2^ix^	137.97 (5)
Al4^vii^—Al3—Al4^ix^	90.02 (6)	Al2—Fe1—Al2^ix^	136.22 (4)
Ni1—Al3—Al4	92.66 (6)	Al4^iii^—Fe1—Al2^ix^	100.29 (6)
Ni1^vii^—Al3—Al4	54.81 (4)	Al5^i^—Fe1—Al4^ix^	107.72 (4)
Al4^viii^—Al3—Al4	59.35 (6)	Al1^xii^—Fe1—Al4^ix^	138.31 (6)
Al2^ix^—Al3—Al4	148.10 (7)	Al1—Fe1—Al4^ix^	133.30 (5)
Al1^xi^—Al3—Al4	120.99 (7)	Al2—Fe1—Al4^ix^	127.69 (5)
Al4^vii^—Al3—Al4	146.70 (7)	Al4^iii^—Fe1—Al4^ix^	66.99 (6)
Al4^ix^—Al3—Al4	97.79 (5)	Al2^ix^—Fe1—Al4^ix^	67.39 (5)
Ni1—Al3—Al2^vii^	115.77 (6)	Ni1^vi^—Al5—Ni1^xii^	180.00 (4)
Ni1^vii^—Al3—Al2^vii^	54.27 (4)	Ni1^vi^—Al5—Al1^xi^	122.66 (3)
Al4^viii^—Al3—Al2^vii^	106.69 (6)	Ni1^xii^—Al5—Al1^xi^	57.34 (3)
Al2^ix^—Al3—Al2^vii^	88.35 (6)	Ni1^vi^—Al5—Al1^v^	57.34 (3)
Al1^xi^—Al3—Al2^vii^	112.61 (6)	Ni1^xii^—Al5—Al1^v^	122.66 (3)
Al4^vii^—Al3—Al2^vii^	57.80 (5)	Al1^xi^—Al5—Al1^v^	180.0
Al4^ix^—Al3—Al2^vii^	59.90 (5)	Ni1^vi^—Al5—Al4^viii^	58.15 (3)
Al4—Al3—Al2^vii^	98.68 (6)	Ni1^xii^—Al5—Al4^viii^	121.85 (3)
Al4^viii^—Si3—Al2^ix^	147.55 (7)	Al1^xi^—Al5—Al4^viii^	65.28 (4)
Al4^viii^—Si3—Al1^xi^	64.16 (5)	Al1^v^—Al5—Al4^viii^	114.72 (4)
Al2^ix^—Si3—Al1^xi^	83.61 (6)	Ni1^vi^—Al5—Al4^xiii^	121.85 (3)
Al4^viii^—Si3—Al4^vii^	102.15 (5)	Ni1^xii^—Al5—Al4^xiii^	58.15 (3)
Al2^ix^—Si3—Al4^vii^	61.35 (5)	Al1^xi^—Al5—Al4^xiii^	114.72 (4)
Al1^xi^—Si3—Al4^vii^	60.05 (5)	Al1^v^—Al5—Al4^xiii^	65.28 (4)
Al4^viii^—Si3—Al4^ix^	153.13 (6)	Al4^viii^—Al5—Al4^xiii^	180.00 (3)
Al2^ix^—Si3—Al4^ix^	59.07 (5)	Ni1^vi^—Al5—Al1^xii^	123.15 (3)
Al1^xi^—Si3—Al4^ix^	141.07 (7)	Ni1^xii^—Al5—Al1^xii^	56.85 (3)
Al4^vii^—Si3—Al4^ix^	90.02 (6)	Al1^xi^—Al5—Al1^xii^	75.622 (19)
Fe1—Si3—Al4	92.66 (6)	Al1^v^—Al5—Al1^xii^	104.378 (19)
Al4^viii^—Si3—Al4	59.35 (6)	Al4^viii^—Al5—Al1^xii^	117.72 (4)
Al2^ix^—Si3—Al4	148.10 (7)	Al4^xiii^—Al5—Al1^xii^	62.28 (4)
Al1^xi^—Si3—Al4	120.99 (7)	Ni1^vi^—Al5—Al1^vi^	56.85 (3)
Al4^vii^—Si3—Al4	146.70 (7)	Ni1^xii^—Al5—Al1^vi^	123.15 (3)
Al4^ix^—Si3—Al4	97.79 (5)	Al1^xi^—Al5—Al1^vi^	104.378 (19)
Al4^viii^—Si3—Al2^vii^	106.69 (6)	Al1^v^—Al5—Al1^vi^	75.622 (19)
Al2^ix^—Si3—Al2^vii^	88.35 (6)	Al4^viii^—Al5—Al1^vi^	62.28 (4)
Al1^xi^—Si3—Al2^vii^	112.61 (6)	Al4^xiii^—Al5—Al1^vi^	117.72 (4)
Al4^vii^—Si3—Al2^vii^	57.80 (5)	Al1^xii^—Al5—Al1^vi^	180.00 (3)
Al4^ix^—Si3—Al2^vii^	59.90 (5)	Ni1^vi^—Al5—Al2^xiv^	122.74 (3)
Al4—Si3—Al2^vii^	98.68 (6)	Ni1^xii^—Al5—Al2^xiv^	57.26 (3)
Ni1^vii^—Al4—Ni1^vi^	113.01 (6)	Al1^xi^—Al5—Al2^xiv^	64.94 (4)
Ni1^vii^—Al4—Al3^viii^	93.58 (6)	Al1^v^—Al5—Al2^xiv^	115.06 (4)
Ni1^vi^—Al4—Al3^viii^	56.57 (5)	Al4^viii^—Al5—Al2^xiv^	91.48 (4)
Ni1^vii^—Al4—Al5^x^	54.30 (3)	Al4^xiii^—Al5—Al2^xiv^	88.52 (4)
Ni1^vi^—Al4—Al5^x^	128.81 (6)	Al1^xii^—Al5—Al2^xiv^	113.79 (4)
Al3^viii^—Al4—Al5^x^	73.87 (5)	Al1^vi^—Al5—Al2^xiv^	66.21 (4)
Ni1^vii^—Al4—Al4^viii^	57.02 (5)	Ni1^vi^—Al5—Al2	57.26 (3)
Ni1^vi^—Al4—Al4^viii^	55.99 (5)	Ni1^xii^—Al5—Al2	122.74 (3)
Al3^viii^—Al4—Al4^viii^	63.53 (6)	Al1^xi^—Al5—Al2	115.06 (4)
Al5^x^—Al4—Al4^viii^	92.61 (7)	Al1^v^—Al5—Al2	64.94 (4)
Ni1^vii^—Al4—Al2	120.96 (6)	Al4^viii^—Al5—Al2	88.52 (4)
Ni1^vi^—Al4—Al2	55.83 (5)	Al4^xiii^—Al5—Al2	91.48 (4)
Al3^viii^—Al4—Al2	111.57 (7)	Al1^xii^—Al5—Al2	66.21 (4)
Al5^x^—Al4—Al2	173.66 (7)	Al1^vi^—Al5—Al2	113.79 (4)
Al4^viii^—Al4—Al2	87.21 (7)	Al2^xiv^—Al5—Al2	180.0

Symmetry codes: (i) −*x*, *y*+1/2, −*z*+1/2; (ii) *x*, *y*+1, *z*; (iii) −*x*+1, *y*+1/2, −*z*+1/2; (iv) −*x*+1, −*y*+1, −*z*; (v) −*x*, −*y*+1, −*z*; (vi) *x*, −*y*+1/2, *z*−1/2; (vii) −*x*+1, *y*−1/2, −*z*+1/2; (viii) −*x*+1, −*y*, −*z*; (ix) *x*, −*y*+1/2, *z*+1/2; (x) *x*+1, *y*, *z*; (xi) *x*, *y*−1, *z*; (xii) −*x*, *y*−1/2, −*z*+1/2; (xiii) *x*−1, *y*, *z*; (xiv) −*x*, −*y*, −*z*.
