# Supplementary material for: Crystal structure of Al8.77Fe0.80Ni1.20Si0.23
Source: IUCrdata. 2026 Mar 19;11(Pt 3):x260266. doi: 10.1107/S241431462600266X (PMC13055986; doi:10.1107/S241431462600266X)
Supplement: Supplementary file 3 [file x-11-x260266-sup3.docx]

**SUPPLEMENTARY MATERIALS:**

**Crystal structure of Al_8.77_Fe_0.80_Ni_1.20_Si_0.23_**

**Mei Chen^a^, Chang zeng Fan^ab*^, Bin Wen^a^ and Lifeng Zhang^ac^**

^a^ State Key Laboratory of Metastable Materials Science and Technology, Yanshan University,

Qinhuangdao 066004, People’s Republic of China

^b^ Hebei Key Lab for Optimizing Metal Product Technology and Performance, Yanshan University, Qinhuangdao, Hebei 066004, People’s Republic of China

^c^ School of Mechanical and Materials Engineering, North China University of Technology, Beijing,100144, People’s Republic of China

*Correspondence email: [chzfan@ysu.edu.cn](mailto:chzfan@ysu.edu.cn)

The chemical compositions were examined quantitatively by energy dispersive X-ray spectroscopy (EDX) analysis attached to a Hitachi S-3400N SEM for the purpose of guiding the crystal structure refinement. The examined points and areas are designated in Figure S1, and the corresponding EDX spectrums as well as the resulting chemical composition are shown in Figure S2 and Table S1, respectively. The deviation relative to the results of refinement of chemical composition is probably caused by the tilt of the single crystal surface to the incident beam. In addition, the conductive adhesives and glues may also result in the detected impurity elements of carbon. However, the presence of all elements of this phase in some parts of the scan proves the existence of this phase. Owing to the coexistence of multiple crystalline phases, a slight deviation is observed between the atomic ratios determined by elemental mapping and those of the crystalline phases; however, such deviation falls within an acceptable and reasonable range. For ease of reading, the atomic ratio of Al, Fe, Ni and Si was calculated and shown in the last column of Table S1.


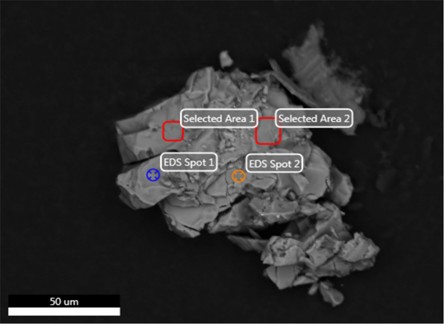


Fig. S1 Single crystal of Al_8.77_Fe_0.80_Ni_1.20_Si_0.23_ with selected spots and areas for EDX analysis

| **Selected Area 1** |
| --- |
| 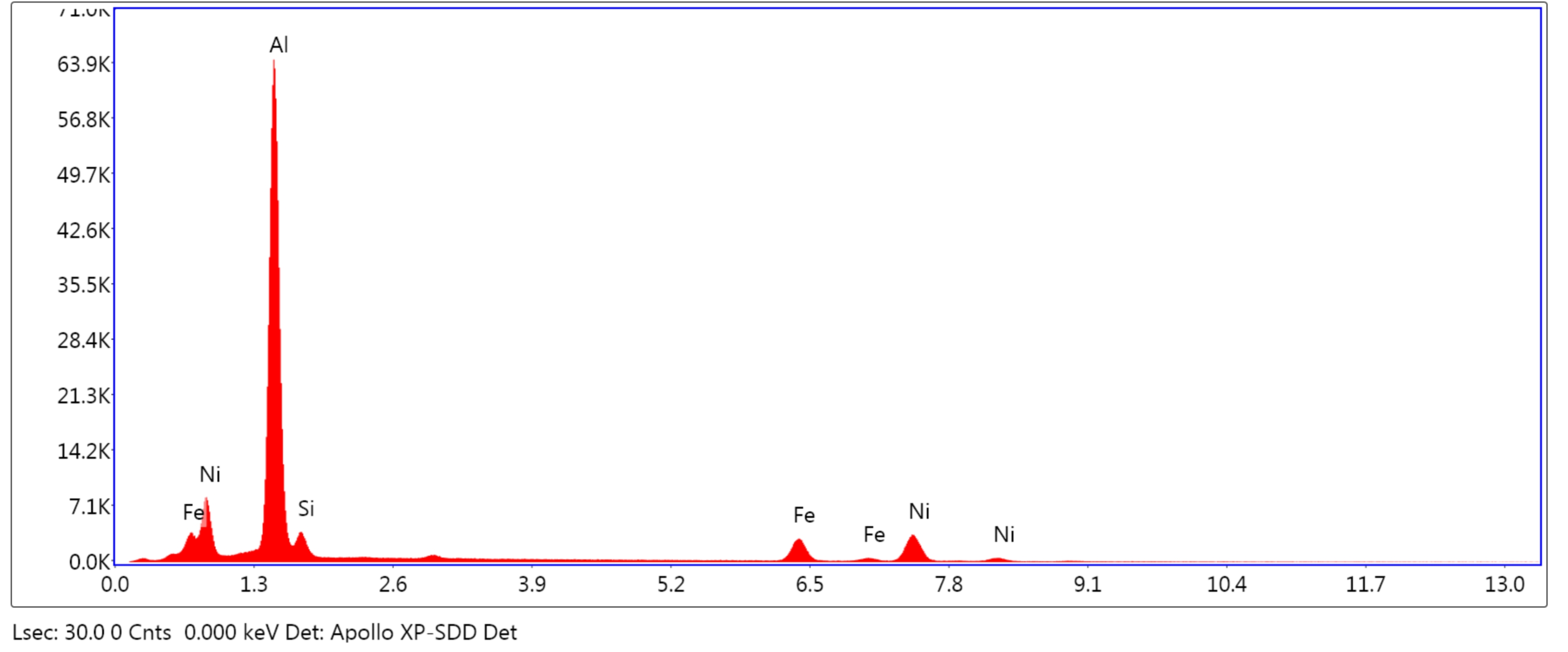 |
|  |
|  |

| **Selected Area 2** |
| --- |
| 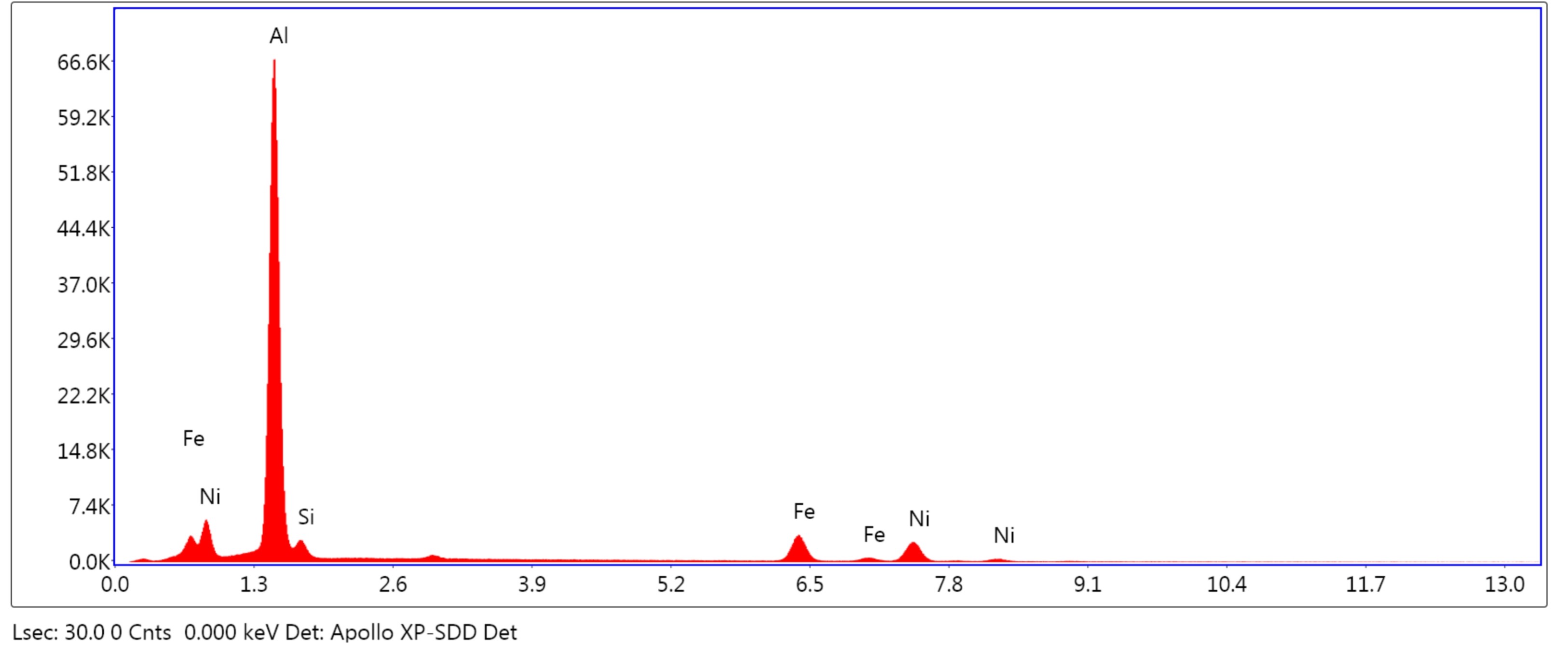 |
|  |
|  |
| **EDS Spot 1** |
| 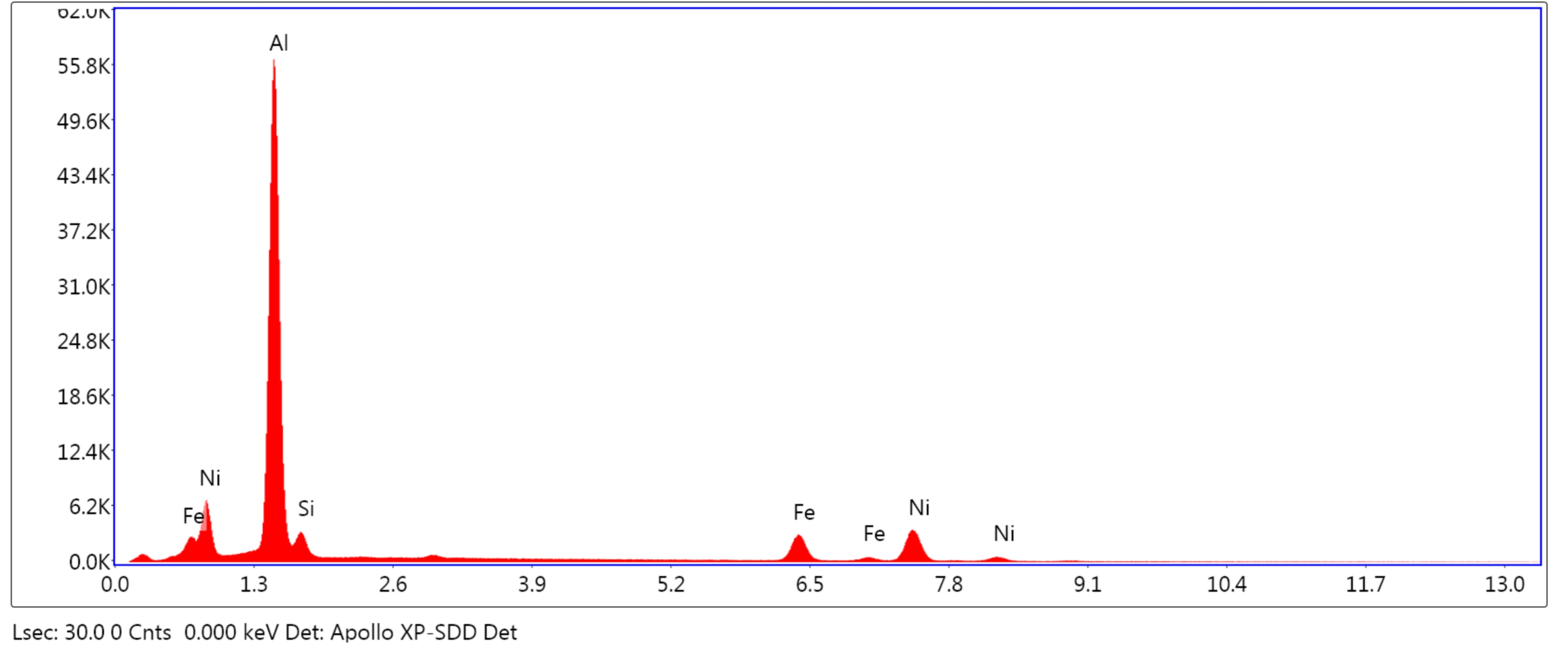 |
|  |
|  |

| **EDS Spot 2** |
| --- |
| 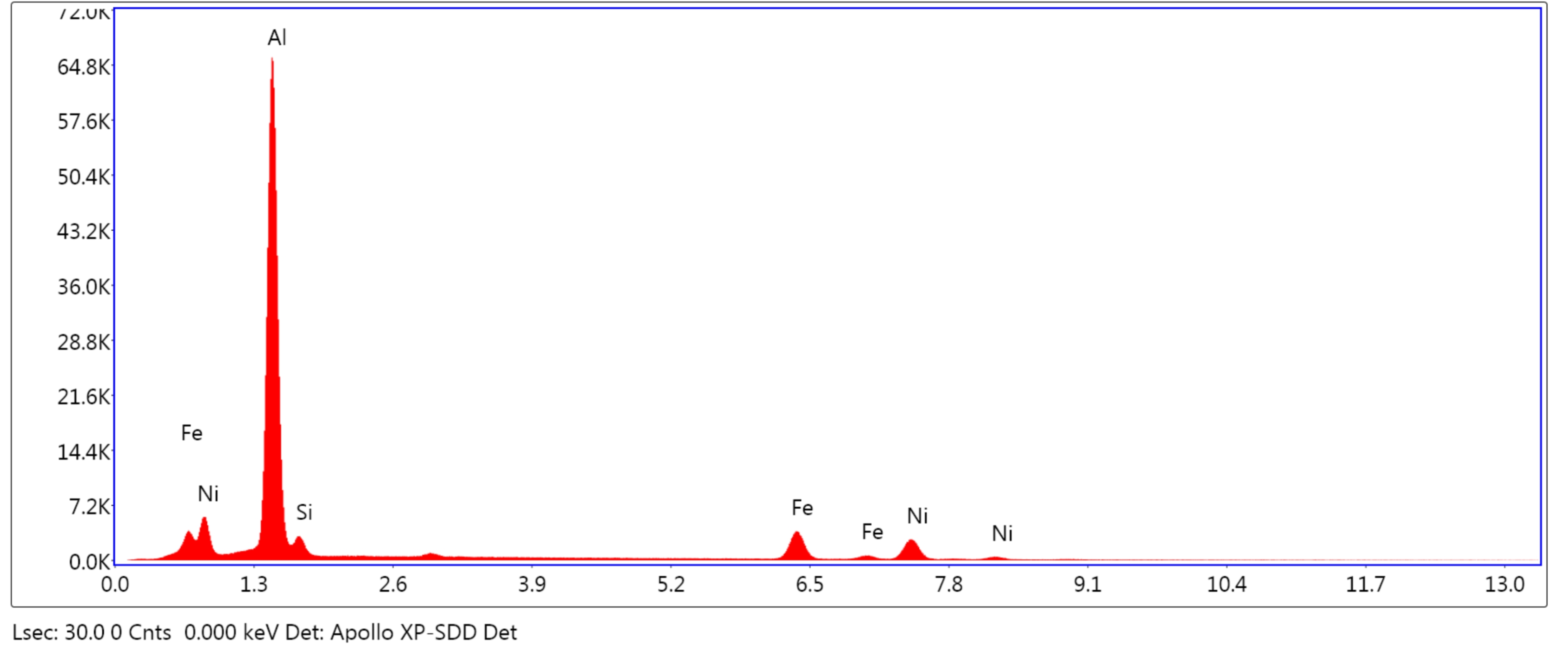 |
|  |
|  |

Fig. S2 The EDX spectrum of the single crystal of Al_8.77_Fe_0.80_Ni_1.20_Si_0.23_ for selected spots and areas

**Table S1 EDX results for selected points and areas as designated in Fig. S1**

|  | Element | Weight (%) | Atomic (%) | Error (%) |
| --- | --- | --- | --- | --- |
| Spot1 | Al | 61.57 | 74.62 | 5.21 |
|  | Si | 6.01 | 7.00 | 7.89 |
|  | Fe | 11.37 | 6.65 | 2.89 |
|  | Ni | 21.05 | 11.72 | 2.46 |
| Spot2 | Al | 65.39 | 77.49 | 4.84 |
|  | Si | 5.50 | 6.27 | 7.93 |
|  | Fe | 13.99 | 8.01 | 2.60 |
|  | Ni | 15.12 | 8.23 | 2.66 |
| Area1 | Al | 63.78 | 75.97 | 4.97 |
|  | Si | 6.54 | 7.49 | 7.86 |
|  | Fe | 10.59 | 6.09 | 2.91 |
|  | Ni | 19.09 | 10.45 | 2.52 |
| Area2 | Al | 66.39 | 78.30 | 4.78 |
|  | Si | 5.26 | 5.96 | 8.04 |
|  | Fe | 13.36 | 7.61 | 2.69 |
|  | Ni | 15.00 | 8.13 | 2.65 |
